# The Mucin MUC4 and Its Membrane Partner ErbB2 Regulate Biological Properties of Human CAPAN-2 Pancreatic Cancer Cells *via* Different Signalling Pathways

**DOI:** 10.1371/journal.pone.0032232

**Published:** 2012-02-29

**Authors:** Nicolas Jonckheere, Nicolas Skrypek, Johann Merlin, Anne Frédérique Dessein, Patrick Dumont, Emmanuelle Leteurtre, Ann Harris, Jean-Luc Desseyn, Christiane Susini, Frédéric Frénois, Isabelle Van Seuningen

**Affiliations:** 1 Inserm, UMR837, Jean Pierre Aubert Research Center, Team #5 “Mucins, epithelial differentiation and carcinogenesis”, Lille, France; 2 Université Lille Nord de France, Lille, France; 3 CNRS UMR8161, Institut de Biologie de Lille, Lille, France; 4 Centre de Biologie-Pathologie, Centre Hospitalier Régional et Universitaire de Lille, Lille, France; 5 Human Molecular Genetics Program, Children's Memorial Research Center, and Department of Pediatrics. Northwestern University, Chicago, Illinois, United States of America; 6 Inserm U995, Lille, France; 7 Inserm, U858, Institut de Médecine Moléculaire de Rangueil, BP84225, Toulouse, France; Sun Yat-sen University Medical School, China

## Abstract

The mucin MUC4 and its membrane partner the ErbB2 oncogenic receptor are potential interacting partners in human pancreatic tumour development. However, the way they function is still largely unknown. In this work, we aimed to identify the cellular mechanisms and the intracellular signalling pathways under the control of both ErbB2 and MUC4 in a human pancreatic adenocarcinomatous cell line. Using co-immunoprecipitation and GST pull-down, we show that MUC4 and ErbB2 interact in the human pancreatic adenocarcinomatous cell line CAPAN-2 *via* the EGF domains of MUC4. Stable cell clones were generated in which either MUC4 or ErbB2 were knocked down (KD) by a shRNA approach. Biological properties of these cells were then studied *in vitro* and *in vivo*. Our results show that ErbB2-KD cells are more apoptotic and less proliferative (decreased cyclin D1 and increased p27kip1 expression) while migration and invasive properties were not altered. MUC4-KD clones were less proliferative with decreased cyclin D1 expression, G1 cell cycle arrest and altered ErbB2/ErbB3 expression. Their migration properties were reduced whereas invasive properties were increased. Importantly, inhibition of ErbB2 and MUC4 expression did not impair the same signalling pathways (inhibition of MUC4 expression affected the JNK pathway whereas that of ErbB2 altered the MAPK pathway). Finally, ErbB2-KD and MUC4-KD cells showed impaired tumour growth *in vivo*. Our results show that ErbB2 and MUC4, which interact physically, activate different intracellular signalling pathways to regulate biological properties of CAPAN-2 pancreatic cancer cells.

## Introduction

Pancreatic Ductal Adenocarcinoma (PDAC) is the 4th leading cause of death by cancer in the world. The survival curve is extremely short (6 months) and the survival rate at 5 years is very low (3%). This dramatic outcome is related to a lack of therapeutic tools and early diagnostic markers which makes pancreatic cancer the most deadly cancer. At the time of diagnosis, more than 80% of PDAC are already metastatic or locally advanced and only about 10 to 15% of patients are considered eligible for surgical resection. Pancreatic carcinogenesis follows a metaplasia/dysplasia/cancer progression with PDAC developing from ductal lesion precursors called Pancreatic Intraepithelial Neoplasia (PanIN-1A/-1B/-2/-3) in which histologic, cytologic and genetic alterations accumulate [1], [2]. Identification of new molecular targets, especially those with altered expression in early PanIN, and deciphering the molecular mechanisms underlying the disease, will undoubtedly allow the development of new therapeutic approaches to stop/slow down tumour progression.

In this context, the membrane-bound mucin MUC4, which is expressed as early as in PanIN-1A whereas it is not expressed in the healthy pancreas, and its membrane partner the oncogenic receptor ErbB2 which is frequently overexpressed in pancreatic cancer as well as in PanINs, represent promising therapeutic targets [3], [4], [5], [6].

The MUC4 gene encodes a large apomucin (930 kDa) composed of two subunits MUC4α and MUC4β [7], [8], [9], [10]. MUC4α is the extracellular subunit featuring a typical hyperglycosylated region. MUC4β is the trans-membrane subunit containing three EGF-like domains (EGF3, EGF1 and EGF2 located from C-terminus to N-terminus) that are conserved in humans and rodents [4], [11]. Experimental evidence with rat homologues of Muc4 and ErbB2 suggests that the EGF-like domains play a role in receptor-ligand interactions and are a regulator in signalling related to growth, motility or differentiation of the cell [4], [12]. From these data, Carraway and collaborators have proposed Muc4 as a modulator of the proliferation/differentiation balance [13] but at this time, such proposed mechanism has not been validated for human MUC4.

The oncogene neu encodes ErbB2/HER2 type I transmembrane growth factor receptor that belongs to the epidermal growth factor receptor (EGFR/ErbB1) family comprising ErbB3 and ErbB4. The ErbB2 protein consists in an extracellular domain, a transmembrane domain and an intracellular tyrosine kinase domain. ErbB2 has no known ligand and is described as a co-receptor which hetero-dimerizes with the other ErbB receptors [14]. ErbB2 is commonly overexpressed in cancers, including PDAC, where ErbB2 is frequently amplified.

Studies in human cells remain scarce and suggest so far a possible role for MUC4 in the biological properties of pancreatic cancer cells [15], [16]. Regarding ErbB2, only one recent study in another pancreatic cancer cell model has shown that ErbB2 may be involved in the properties of pancreatic cancer cells [17]. Previous works in colon and pulmonary cells showed that MUC4 and ErbB2 may act as a functional complex and transduce signals intracellularly [18], [19]. However, it remains unclear whether MUC4 and ErbB2 activate the same signalling pathway(s).

In the present work, we undertook to identify the intracellular signalling pathways under the control of either ErbB2 or MUC4 in the same cellular model of pancreatic cancer to test this hypothesis. We show that human MUC4 and ErbB2 do physically interact in pancreatic cancer cells and that inhibition of expression of each membrane partners does not impair the same signalling pathways (inhibition of MUC4 expression affects the JNK pathway whereas that of ErbB2 alters the MAPK pathway). Different effects on the biological properties of pancreatic cancer cells were observed, suggesting independent activities of these two proteins with a role in pancreatic tumour growth for ErbB2, while MUC4 appears to be involved in both tumour growth and dissemination.

## Materials and Methods

### Establishment of ErbB2 and MUC4 KD cell lines

The CAPAN-2 pancreatic cancer cell line (ATCC, HTB-80) was cultured as previously described [20]. ErbB2-KD cells were obtained following stable transfection of CAPAN-2 cells with pGeneClip™ puromycin vector encoding ErbB2 ShRNA (SA Biosciences™). Stable transfection of 1 µg of ScaI digested plasmid was performed with Effectene® (Qiagen, Courtaboeuf, France) following manufacturer's protocol. The empty vector was used to raise control clones called Non Targeting (NT). Selection was performed using puromycine (0.1 µg/ml, InvivoGen, Limoges, France) and clones were isolated by limited serial dilution. MUC4-Knocked Down (KD) cells were obtained by retroviral infection of CAPAN-2 cells with pRetroSuper plasmid (SA Biosciences™) containing a sequence targeting MUC4 (5′-AAGTGGAACGAATCGATTCTGTTCAAGAGACAGAATCGATTCGTTCCACTT-3′). The empty vector was used to raise control clones called *Mock*. Selection was performed using G418/geneticine (300 µg/ml, Invitrogen, Cergy Pontoise, France) and clones were isolated by serial limit dilution.

### Western blotting

Cytosolic, nuclear and total cellular extracts were prepared as described in Van Seuningen *et al.* [21] and Jonckheere *et al.* [22], respectively and kept at −80°C until use. Protein content (2 µl of nuclear extracts) was measured in 96-well plates using the bicinchoninic acid method as described in the manufacturer's instruction manual (Pierce). Western blotting was carried out on nitrocellulose membrane (0.2 µm, Schleicher and Schuell) as previously described [23]. Membranes were probed with antibodies against ErbB-2 (clone Ab-1, dilution 1/500), p27kip1 (dilution 1/500) from Lab Vision Neomarker, USA; phospho-p42/44MAPK (Thr202/Tyr204) (clone 20G11, dilution 1/500), p42/44MAPK (clone I37F5, dilution 1/500), phospho-SAPK/JNK (Thr183/Tyr185) (#9251, dilution 1/500), SAPK/JNK (clone 56G8, 1/500), phospho-p38MAPK (Thr180/Tyr182) (clone D3F9, 1/1000), p38MAPK (#9212, dilution 1/1000), FAK (#3285, dilution 1/1000), phospho-Akt (Ser473) (clone D9E, 1/1000), Akt (clone C67E7, dilution 1/1000), cyclin D1 (clone DCS6, dilution 1/500), EGFR (#2232, dilution 1/500), all from Cell Signaling Technology, USA; ErbB-3 (clone C-17, 1/500), ErbB-4 (clone C-18, dilution 1/500), MMP2 (clone H-76, dilution 1/500), MMP9 (clone 6-6B, dilution 1/500), Bcl-xL (clone H-5, dilution 1/500), Bax (clone N-20, dilution 1/500), MUC4 (8G7, dilution 1/500) all from Santa Cruz Biotechnology, USA; MUC1 (M8, generous gift from Dr D. Swallow, London), or β-actin (A5441, dilution 1/5000) from Sigma, France. Antibodies were diluted in Tris-Buffered Saline containing 5% (w/v) non-fat dry milk and Tween-20 (TBS-T), except for MUC4 and β-actin and incubated overnight at 4°C before processing with immunostaining. Peroxidase-conjugated secondary antibodies (Sigma) were used and immunoreactive bands were visualised using the West Pico chemoluminescent substrate (Perbio, Brebières, France). Chemo-luminescence was visualised using LAS4000 apparatus (Fujifilm) and results were integrated using Gel analyst software® (Claravision). The data presented are representative of three independent experiments.

### Co-immunoprecipitation of the MUC4-ErbB2 complex

150 µg of CAPAN-2 cellular extract were incubated overnight with 1.5 µg of anti-ErbB2 antibody (Rabbit polyclonal, Ab-1, Thermo Scientific). Protein A was covalently attached to crosslinked 4% agarose beads (Sigma, France), equilibrated with 1× binding buffer (200 mM Tris-HCl pH 7.5 containing 1 M NaCl, 20 mM EDTA, and 2% NP40 (v/v)) and added to the lysate-antibody mix and incubated on a rotating platform for 2 h at 4°C. Beads were then washed three times with 1× binding buffer. Rabbit IgGs (Millipore) were used as a negative control. Washed beads were then mixed with 2× SDS gel loading buffer before electrophoresis on a 2% (w/v) agarose gel and immunoblotting as described before [23].

### Construction of the GST-MUC4_EGF3+1+2_ fusion protein

The cDNA encoding the MUC4_EGF3+1+2_ sequence was amplified by PCR using a FLAG epitope-tagged version of the MUC4β subunit [16] called MUC4F2-CF2 as the template and F_EGF3 (5′-CGCGGATCCGCCTGTGAGGAGCCG-3′) and R_EGF1 (5′-TCCCCCGGG TCAGAAGCAGCGGCTGTC-3′) as primers. The PCR product encoding MUC4_EGF3+1+2_ was digested by *BamHI* and *SmaI* and inserted into pGEX-4T1 (GE Lifesciences). The pGEX-4T1-GST-MUC4_EGF3+1+2_ construct was then transfected in B834pLysS *E. coli* (Novagen) using electroporation and *E. coli* was grown in Luria Bertani medium (Invitrogen) to an OD_600_ of 0.8. GST-MUC4_EGF3+1+2_ fusion protein expression was then induced by adding 1 mM of isopropylthiogalactopyranosyl (Ambion) at 15°C overnight. The cells were harvested by centrifugation at 3800× g at 4°C, resuspended in 60 ml lysis buffer (1× PBS, 1 mM DTT, 1 mM EDTA, 1% (v/v) Triton X/100) and lysed by sonication (Branson Sonifier 250). After centrifugation (20 000× g, 90 min, 4°C), the supernatant was recovered, and GST-MUC4_EGF3+1+2_ was separated from the whole-cell lysate using glutathione agarose beads (Qiagen). After washing beads with lysis buffer, GST-MUC4_EGF3+1+2_ fusion protein was eluted by 40 mM of reduced glutathione in a 50 mM Tris-HCl pH 8.0 buffer containing 150 mM NaCl, 0.1% (v/v) Triton X/100 and 1 mM DTT. Protein purity was determined by Coomassie blue staining after a 12% SDS-PAGE. The purified protein was dialysed against 1× binding buffer and stored at 4°C until use.

### GST pull-down

The GST-MUC4_EGF3+1+2_ was loaded on equilibrated glutathione beads (Qiagen,) as described by the manufacturer. After overnight binding at 4°C in the presence of the human recombinant ErbB2 protein (5 µg, R&D systems, Lille, France), the glutathione beads were washed 3 times with 1× binding buffer. The washed beads were then mixed with 2× SDS loading buffer and boiled at 100°C for 5 min. The supernatant was loaded on a 6% SDS-PAGE and blotted on a PVDF membrane. ErbB2 Western blotting was carried out as described above.

### Proximity ligand assay

*In situ* proximity ligand assays were performed using Duolink II Red Starter Kit (Olink, UPPSALA, Sweden) following the manufacturer's protocol. Briefly, 2.5×10^5^ cells were seeded in a Chamber Slide permanox (Nunc, Brumath, France) and incubated 72 h to reach 70–80% confluence. Cells were fixed 20 min with 4% (v/v) paraformaldehyde at RT before a blocking step with the blocking solution for 30 min at 37°C. Primary antibodies against MUC4 (8G7) and ErbB2 (C-18) were diluted at 1/50 in 1× PBS pH 7.4 and incubated for 90 min at RT. PLA probe anti-rabbit PLUS and anti-mouse MINUS were diluted at 1/5 in antibody diluent, and incubated with cells 1 h at 37°C after two washing steps. Ligation and amplification were then performed at 37°C in order to visualize the complex. Slides were mounted with Duolink II Mounting Medium containing DAPI. Stainings were visualized with a Zeiss LSM 710 confocal microscope (Zeiss, Jena, Germany), images were captured and analysed with the Zeiss Efficient Navigation software (Zeiss, Jena, Germany).

### Confocal microscopy

Confocal microscopy was carried out as previously described [24]. MUC4 (8G7, Santa Cruz) and ErbB2 (C-18, Santa Cruz) antibodies were used at the 1/50 dilution in 1× D-PBS+Mg^2+^+Ca^2+^ (Invitrogen) containing 0.2% (w/v) saponin and 2% (v/v) goat serum. AlexaFluor® 594 goat anti-mouse and AlexaFluor® 488 goat anti-rabbit (Invitrogen) secondary antibodies were respectively used to detect MUC4 and ErbB2 expression.

### Migration and Invasion assays

Cell migration and invasion properties of the different clones were assessed using respectively 24 well control Boyden chambers (8 µm pores) and chambers coated with Matrigel® matrix (BD Biosciences, le Pont de Claix, France) following manufacturer's protocol. Briefly, 10% (v/v) foetal bovine serum was used as chemoattractant in the lower chamber. 5×10^4^ cells were plated in the top chamber and incubated for 48 h. After staining with DiffQuick (Mediane Diagnostics, Plaisir, France), cells on the lower surface were counted using light microscopy at ×100 magnification. Eight random vision fields were counted and the experiment was repeated four times.

### Flow Cytometry

Cells were cultured during 24 h before being harvested by trypsinization, and then resuspended in 1× PBS. The cells were fixed by addition of 1 ml of 70% (v/v) ethanol and incubation on ice for 30 min. Cells were then washed with 1× PBS, treated for 5 min with RNase A (100 mg.ml^−1^) and finally stained with propidium iodide (50 mg.ml^−1^, Sigma) for 30 min. Cell analysis was carried out on a Beckman Coulter EPICS XL3-MCL (Villepinte, France) using the Wincycle software (Phoenix Flow Systems, San Diego, CA, USA).

### Subcutaneous xenografts

Subcutaneous (SC) xenografts (6×10^6^ cells in 150 µl of RPMI 1640) of CAPAN-2 clones were injected with 150 µl of Matrigel® (ref 354262, BD Biosciences, le Pont de Claix, France) into severe-combined immunodeficient (SCID) mice that were bred and maintained under pathogen-free conditions (6 mice/cell type). Tumour development was followed periodically. The tumour volume (mm^3^) was determined by calculating V = W^2^×L/2 in which W corresponds to the width (in mm) and L to the tumour length (in mm). Mice were killed 55 days after inoculation. All procedures were in accordance with the guideline and approved by the animal care committee (Comité Ethique Expérimentation Animale Nord Pas-de-Calais, Permit/Protocol number: AF042008). For each SC tumour, tissue was fixed in formalin before paraffin inclusion. Mice presenting features of pain (weight loss, cachexia, piloerection) or bearing tumour reaching 1 cm^3^ were sacrificed.

### Immunohistochemistry

SC xenografted tissues were fixed in 10% (w/v) buffered formaldehyde, embedded in paraffin, cut at 4 µm thickness and applied on SuperFrost® slides (Menzel-Glaser, Braunschweig, Germany). Slides were then stained with Hematoxylin-Eosin-Saffron-Astra blue. Manual immunohistochemistry (IHC) was carried out as described in Van der Sluis *et al* [25] and automatic IHC with an automated immunostainer (ES, Ventana Medical System, Strasbourg, France) as in Mariette *et al* [26]. The antibodies were used as followed: 1∶200 dilution of anti-ErbB2 (DAKO), 1∶100 of anti-MUC4 8G7 (Santa Cruz Biotechnology) and 1∶50 of anti-MUC1 M8.

### DNA Microarray analysis

Comparative transcriptome analyses were conducted on four MUC4-KD and four ErbB2-KD cellular clones. They were compared to a pool of four Mock and four NT cellular clones, respectively. RNA quality was checked using the Agilent 2100 Bioanalyser (Agilent Technologies, Massy, France). cRNA samples were synthesized using low input RNA fluorescent linear amplification kit (Agilent). Hybridization of Cy3 and Cy5 labelled cRNA was performed on the human 44 K pangenomic 60 mer oligonucleotide microarray (Agilent) according to the manufacturer's protocol. Microarrays were scanned using the Agilent scanner G2505C and Feature Extraction software (v10.5). Data were processed with the GeneSpring software (v10) for normalization, filtering, and statistical analysis. The genes upregulated or downregulated (fold change >5) with statistical significance (P<0.05) were sorted using asymptotic P value computation. All data are MIAME compliant. The raw data has been deposited in Gene Expression Omnibus (GEO, accession number: GSE31322).

### qRT-PCR

Total RNAs from pancreatic cancer cells were prepared using the NucleoSpin® RNA II kit (Macherey Nagel) following manufacturer's protocol. cDNAs were prepared as previously described [27]. PCR was performed using SsoFast™ Evagreen Supermix kit following manufacturer protocol using the CFX96 real time PCR system (Biorad). Primer information is given in table S1.

### Statistical analysis

Statistical analyses were performed using Graphpad Prism 4.0 software (Graphpad softwares Inc., La Jolla, USA). Data are presented as mean ±SEM. Differences in the mean of two samples were analysed by the student's *t* test or one way ANOVA test with selected comparison using Tukey's HSD post-hoc test with differences less than 0.05 considered significant and were indicated with an *. ** indicates p<0.01, *** indicates p<0.001. Differences of contingency were analysed using Chi square test.

## Results

### MUC4 and ErbB2 physically interact in the CAPAN-2 pancreatic cancer cells

**Figure 1:**
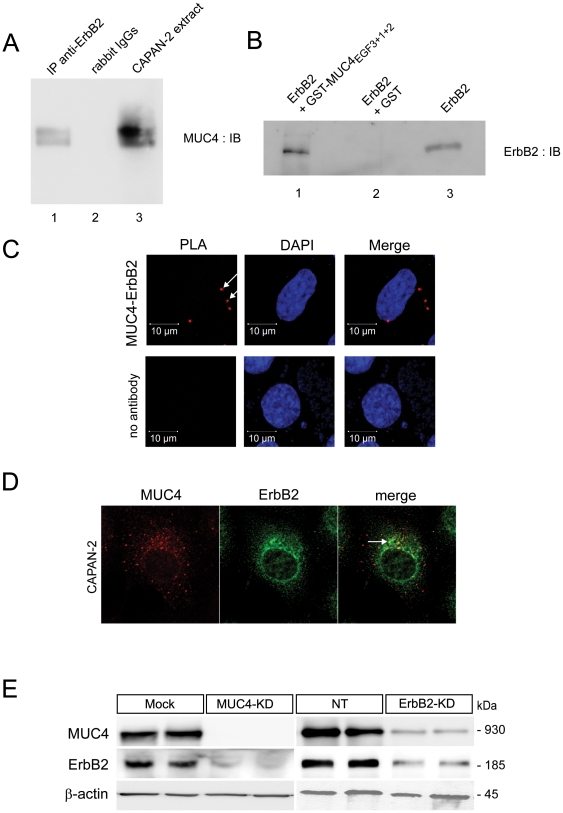
MUC4 and ErbB2 form a complex – Characterisation of MUC4-KD and ErbB2-KD cells. (**A**) Co-immunoprecipitation of 150 µg of CAPAN-2 cellular extract with anti-ErbB2 antibody (lane 1) or rabbit IgGs (lane 2). CAPAN-2 cell extract alone (lane 3). (**B**) ErbB2 immunoblot of GST pull-down of recombinant human ErbB2 protein with GST-MUC4_EGF3+1+2_ glutathione beads (lane 1) or GST glutathione beads (lane 2). Recombinant ErbB2 alone (lane 3). (**C**) *In situ* proximity ligand assay on CAPAN-2 cells. MUC4 and ErbB2 complexes (red dots) are indicated by an arrow. Nuclei were stained using DAPI. Control experiment was conducted in the absence of primary antibody. (**D**) Immunofluorescence detection of MUC4 (red) and ErbB2 (green) by confocal microscopy showed co-localization of the two proteins (yellow). (**E**) Expression of MUC4 and ErbB2 in two representative clones of MUC4-KD and ErbB2-KD cells and their respective controls (Mock and NT) by Western blotting.

Immunoprecipitation of CAPAN-2 cellular extract with anti-ErbB2 antibody was performed before immunoblotting with anti-MUC4 antibody. MUC4 immunostaining indicates that MUC4 co-immunoprecipitated with ErbB2 in CAPAN-2 cells (Figure 1A, lane 1). Specificity of the interaction was assessed using irrelevant rabbit IgGs that showed no immunoprecipitated band (lane 2). In order to show a direct physical interaction between MUC4 and ErbB2, we first carried out a GST pull-down assay. For that, we made a GST-MUC4_EGF3+1+2_ fusion protein which contains the three EGF-like domains of MUC4. We then prepared two affinity columns bearing either the GST-MUC4_EGF3+1+2_ fusion protein or the GST alone on which recombinant human ErbB2 was loaded. Following affinity chromatography, anti-ErbB2 immunoblot showed an ErbB2 specific band for the glutathione beads column bearing the GST-MUC4_EGF3+1+2_ fusion protein (Figure 1B, lane 1). No bands were observed for the glutathione column bearing GST alone (lane 2).

Interaction between MUC4 and ErbB2 was then confirmed using another approach that is the *in situ* proximity ligation assays (PLA). The results indicate that MUC4 and ErbB2 form an endogenous complex in CAPAN-2 cells (red dots, arrows, Figure 1C). Finally, confocal microscopy studies confirmed the co-localisation of MUC4 and ErbB2 in CAPAN-2 cells (Figure 1D). Altogether, these results indicate that MUC4 and ErbB2 physically interact in CAPAN-2 pancreatic cancer cells *via* MUC4_EGF3+1+2_ region that contains the three EGF-like domains. We then undertook to identify the cellular mechanisms and the intracellular signalling pathways under the control of both partners.

### Generation and characterization of stable ErbB2-KD and MUC4-KD cellular clones

The five ErbB2-KD and seven MUC4-KD cellular clones showed respectively almost complete or total inhibition of ErbB2 and MUC4 expression compared to control clones (Figure 1E). Western blot analysis of another membrane-bound mucin that is important in cancer, MUC1, indicated that inhibition of MUC4 dramatically impaired that of MUC1 whereas inhibition of ErbB2 had no effect (Figure S1). When we looked at the expression of the three other members of the ErbB receptor family, inhibition of MUC4 or ErbB2 expression led to a strong decrease of ErbB2 and ErbB3 (MUC4-KD) and of ErbB3 (ErbB2-KD), respectively, whereas no effect was detected for ErbB1 and ErbB4 (Figure S1).

### Role of ErbB2 and MUC4 on cell proliferation and apoptosis

**Figure 2:**
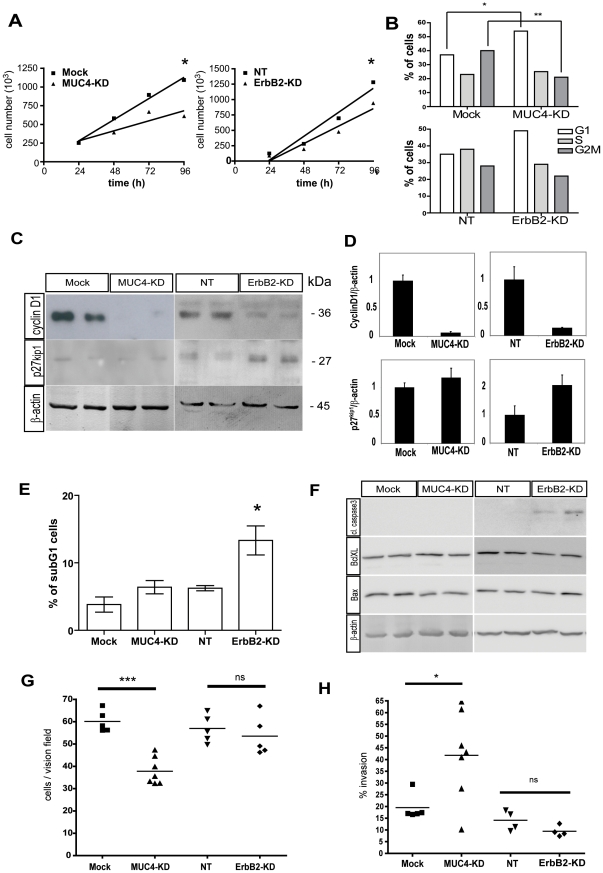
Role of ErbB2 and MUC4 on cell proliferation, cell cycle apoptosis, migration and invasion properties of pancreatic cancer cells. (**A**) Cell growth was assessed by cell counting at 24, 48, 72 and 96 h for CAPAN-2 MUC4-KD or ErbB2-KD and their respective controls (Mock and NT). * = p<0.05 using student *t*-test. (**B**) Cell cycle distribution profiles of MUC4-KD, ErbB2-KD and their control clones (Mock and NT) by flow cytometry following incubation with propidium iodure. The values are expressed as the mean of three independent experiments. * = p<0.05 using Chi square test. ns = non significant. (**C**) Effect of MUC4 or ErbB2 silencing was analysed on cell cycle marker cyclin D1 and p27^kip1^ by western blot. β-actin was measured as the internal control. (**D**) Bands were quantified by densitometry. Histograms of the ratio (cyclin D1 or p27^kip1^/β-actin) are shown. (**E**) % of subG1 population of MUC4-KD, ErbB2-KD and controls (Mock and NT, respectively) was determined by flow cytometry following incubation with propidium iodure (* = p<0.05). (**F**) Western blot were carried out for cleaved caspase 3, Bcl_XL_ and Bax in MUC4-KD, ErbB2-KD and their respective controls (Mock and NT). β-actin was evaluated as an internal control. (**G**) Migration properties of MUC4-KD, ErbB2-KD and control clones (Mock and NT) were evaluated using Boyden chambers. Results are expressed as average migratory cell number per vision field (*** = P<0.001, ns = non significant). (**H**) % of invasion (invasive cells/migratory cells) was determined using Boyden chambers coated with Matrigel®.

MUC4-KD cells showed a decreased proliferation from 48 h that was sustained at 72 h and became significant at 96 h (44% less than Mock cells, p<0.05). ErbB2-KD cells were also less proliferative with a significant decrease of 27% at 96 h (p<0.05) (Figure 2A). A strong cell cycle arrest in the G1 phase (54.5%±0.13) was observed in MUC4-KD cells when compared with Mock cells (37.4%±7.2, *, p = 0.016) (Figure 2B). Moreover, the G2M phase was much shorter in MUC4-KD cells (20.9%±4.7) compared to Mock control cells (39.6%±6.7, **, p = 0.0035) (overall *, p = 0.0102). In ErbB2-KD cells, a trend toward a cell cycle arrest in G1 also occurred that remained statistically non significant (p = 0.119) (Figure 2B). In order to identify the mechanisms responsible for this alteration in proliferation, expression of cell cycle markers was evaluated by Western blotting (Figure 2C). Clearly, decreased proliferation in MUC4-KD cells was associated with repression of cyclin D1. In ErbB2-KD cells, decreased proliferation was associated with both increased expression of p27^kip1^ cell cycle inhibitor and decrease of cyclin D1 (Figure 2D), suggesting a cellular arrest at the G1/S proliferation checkpoint.

To assess whether MUC4 or ErbB2 inhibition could also affect apoptosis, measurement of apoptotic cell population (subG1 cells) by flow cytometry was carried out (Figure 2E). The results indicated a significant increase of subG1 population (13.33%±2.1) in ErbB2-KD cells when compared to NT control clones (6.2%±0.4) (*, p = 0.017). In MUC4-KD cells, no differences in subG1 cell population were observed when compared to Mock control clones (ns, p = 0.19). Expression of apoptotic markers by immunoblotting indicated that Bax and Bcl_XL_ were not altered in both MUC4-KD and ErbB2-KD cells whereas increase of cleaved caspase-3 was observed in ErbB2-KD cells (Figure 2F).

### Role of MUC4 and ErbB2 in cell migration/invasion/adhesion

To assess the role of MUC4 and ErbB2 in cell migration/invasion, experiments were carried out in Boyden chambers without or with Matrigel® coating, respectively. The results indicated that a significant lower number of migratory cells was observed in MUC4-KD cells (37.8±1.5) compared to Mock cells (60.1±3.3) (p≤0.001) whereas no significant difference was found in ErbB2-KD cells (53.5±8.8) compared to NT control clones (56.2±6.3) (Figure 2G). When we measured invasiveness, MUC4-KD cells appeared significantly more invasive (41.8%±1.5, n = 7) than cells expressing MUC4 (mock) (19.5%±1.5, n = 5) (p = 0.029) (Figure 2H). ErbB2-KD clones, on the other hand, were slightly less invasive (9.4%±1.15) than cells expressing ErbB2 (NT, 14.14%±2.1) but this decrease was not significant (p = 0.09). The ability of MUC4-KD and ErbB2-KD cells to adhere on Matrigel® (made of two components of the pancreatic extracellular matrix collagen IV and laminin) or collagen I was also assessed but no significant differences were found for either cells (not shown).

### Role of ErbB2 and MUC4 in intracellular signalling

**Figure 3:**
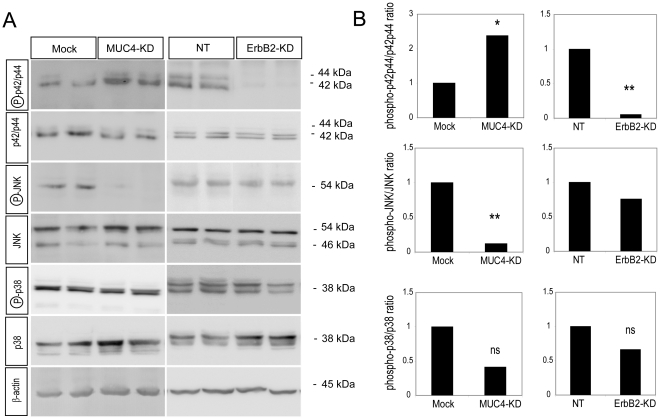
Impact of ErbB2 and MUC4 on MAPK/SAPK (p42/p44, JNK, p38) signalling pathways. (A) Western blot analyses were carried out on cytosolic extract of MUC4-KD, ErbB2-KD and controls (Mock and NT respectively) for expression of both phosphorylated and constitutive forms of p42/p44, JNK and p38 MAPK. β-actin was used as the internal control. Two representative clones are presented. (B) Bands were quantified by densitometry. Histograms of the ratio (phosphorylated/constitutive form) for p42/p44, JNK and p38 MAPK kinases are shown.

The impact of ErbB2 or MUC4 on the major pathways of intracellular signalling was studied by Western blotting for: MAPKs (p42/44, p38 and JNK), Akt, and Focal Adhesion Kinase (FAK) (Figure 3 and Figure S2). Phosphorylation of p42/p44 MAPK was totally abolished in ErbB2-KD cells compared to NT control clones, indicating complete inhibition of this pathway subsequent to ErbB2 silencing. In MUC4-KD cells compared to mock clones, constitutive p42/p44 MAPK decreased whereas phosphorylated p42/44 increased suggesting an activation of the MAPK pathway linked to MUC4 suppression.

When we looked at the JNK pathway, a total repression of JNK phosphorylation was observed in MUC4-KD clones compared to Mock controls suggesting that MUC4 expression dramatically impacts JNK pathway activity. No effect on the JNK pathway was observed in ErbB2-KD clones. p38 MAPK pathway did not appear to be significantly altered following either ErbB2 or MUC4 silencing (Figure 3). Similarly, Akt and FAK pathways were not significantly modified (Figure S2).

Altogether, these results show that loss of MUC4 and ErbB2 dramatically impairs JNK and p42/44 MAPK signalling pathways, respectively.

### Effect of ErbB2 and MUC4 on tumour properties *in vivo*

**Figure 4:**
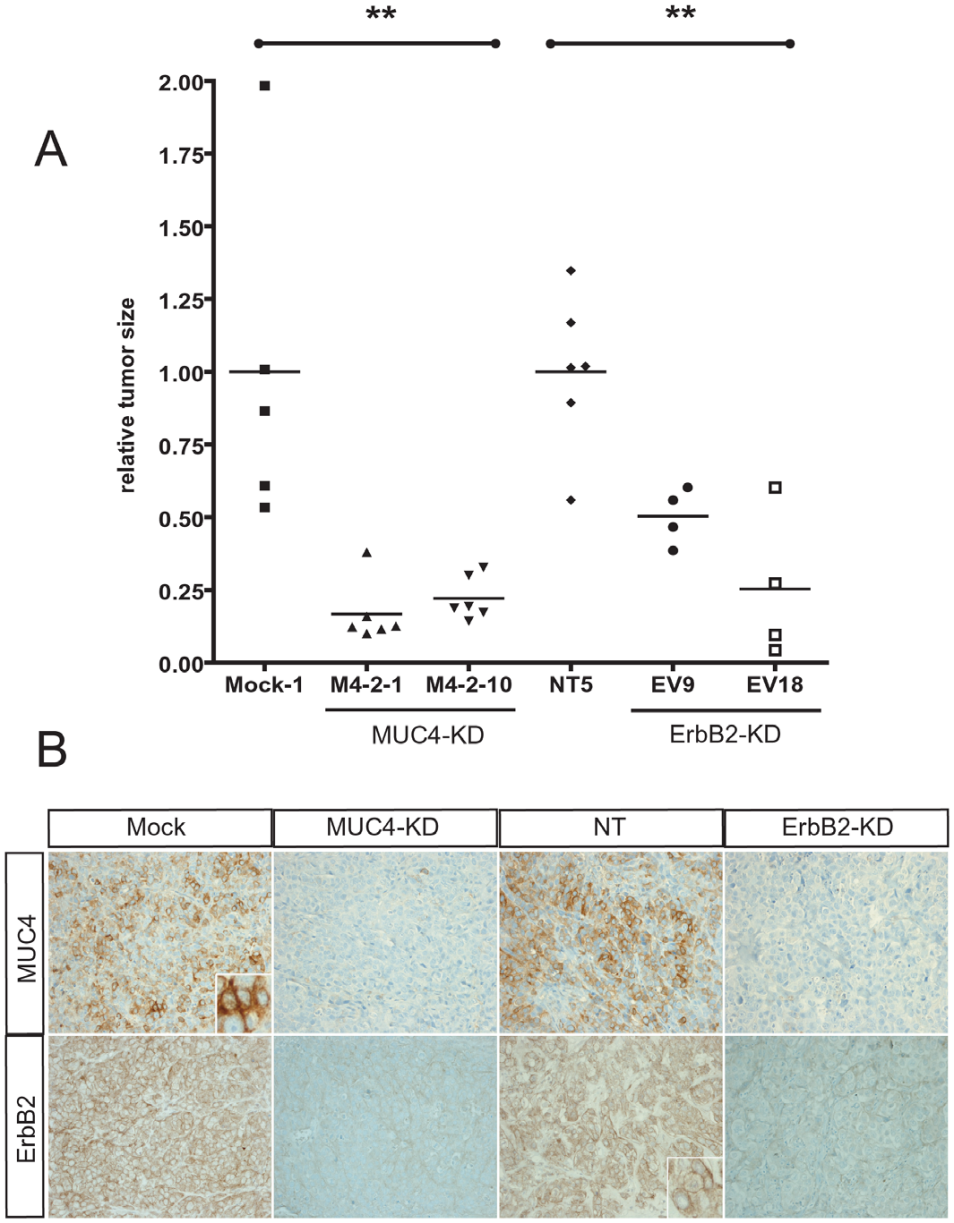
Effect of ErbB2 and MUC4 on tumour growth *in vivo*. (A) Subcutaneous xenograft of MUC4-KD (M4-2-1 and M4-2-10), ErbB2-KD (EV9 and EV18) and control (Mock-1 and NT5) cells were performed in six SCID mice. Relative tumour size was determined at day 22. (B) IHC analysis of MUC4 and ErbB2 expression was analysed on extracted tumours. MUC4 is expressed at the membrane surface and within the cytoplasm in Mock cells (insert). ErbB2 is expressed at the membrane surface in NT control cells (insert).

To confirm *in vitro* data of MUC4 and ErbB2 effects on tumour cell properties, SC xenograft studies were carried out. The results indicate that the tumour volume was significantly lower in xenografted mice with M4-2-1 or M4-2-10 clones at day 22 (Figure 4A) in which absence of MUC4 was confirmed by IHC (Figure 4B). Reduction of ErbB2 expression previously shown *in vitro* by Western blotting was also confirmed *in vivo* in MUC4-KD tumours by IHC (Figure 4B). The relative tumour volume was 0.14±0.05 for M4-2-1 and 0.22±0.03 for M4-2-10 (80–90% decrease) when compared to Mock control tumour volume (1±0.3). The decrease was statistically significant for both clones (**, p = 0.0012) (Figure 4A).

A significant reduction of tumour size was also observed in tumours derived from EV9 (0.55±0.17) and EV18 (0.18±012) ErbB2-KD cells when compared to NT controls (1±0.1, **, p = 0.017). As expected, a dramatic reduction of ErbB2 expression in ErbB2-KD tumours was observed by IHC (Figure 4B). Interestingly, MUC4 expression was strongly repressed in ErbB2-KD tumours (Figure 4B) confirming the strong decrease in MUC4 level in ErbB2-KD cells observed by immunoblotting (see Figure 1).

Altogether, these results indicate that both ErbB2 and MUC4 play a role in pancreatic tumour growth *in vivo*.

### Effect of ErbB2 and MUC4 on gene expression

**Figure 5:**
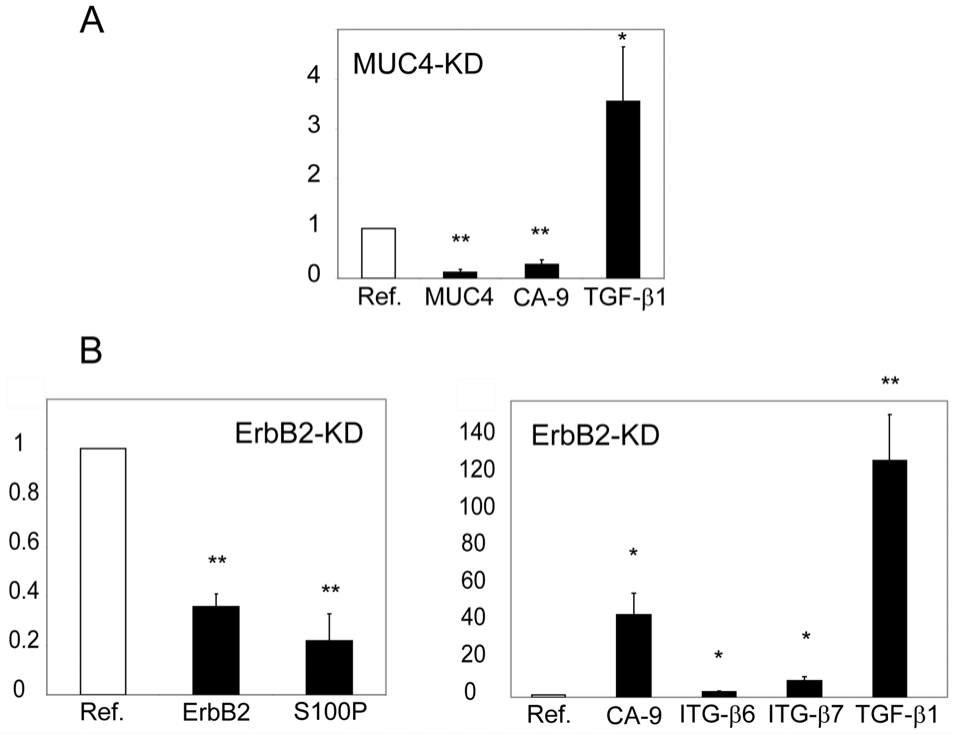
Validation of impact of ErbB2 and MUC4 on gene expression. Levels of expression of selected genes from microarray analysis were studied by qRT-PCR in MUC4-KD clones (**A**) or ErbB2-KD clones (**B**). Expression levels were normalized to mRNA levels of GAPDH housekeeping gene and shown as x-fold relative to the normalized expression of the respective target gene in Mock cells. Relative amounts of target genes were calculated using the ΔΔCt method. Values are means ± SEM from four independent samples. * = p<0.05 using student *t*-test. ** = p<0.01 using student *t*-test.

In order to identify new pathways regulated by ErbB2 and MUC4, comparative transcriptome analyses were carried out by using DNA microarrays on expressing and non-expressing cell lines (table S2 & S3, GEO accession number: GSE31322). In ErbB2-KD cells, 254 and 61 genes were differentially expressed with equal to and/or more than five or two-fold difference, respectively in ErbB2-KD vs. Mock cells (p<0.01). In MUC4-KD cells, over 2282 and 326 genes were altered with a five or two-fold difference, respectively in MUC4-KD vs. Mock cells (Figure S3). Notably, more genes were found down-regulated (n = 209) than up-regulated (n = 117) following MUC4 silencing (fc>5, p<0.01). Interestingly, only 37 (f.c.>2) and 8 (f.c.>5) altered genes were common following ErbB2 and MUC4 silencing (p<0.01). Among those, were found genes encoding ATPase, Ca^2+^ transporting, type 2C, member 2 (ATP2C2) (NM_014861), integrin-β7 (ITG-β7) (NM_000889), keratin81 (NM_002281), keratin86 (NM_002284), Serpina3 (NM_001085), collagen type VIII alpha1 (NM_001850) (f.c.>5) and STAT1 (IFN-γ signalling pathway) (NM_139266) (f.c.>2). Regulated genes in ErbB2-KD cells were related to control of transcription, signal transduction, cell adhesion or induction of apoptosis (table S4). Regulated genes in MUC4-KD cells were related to control of transcription, DNA-dependent transcription, signal transduction, cell cycle, apoptosis or cell adhesion (table S5). Altered gene expression was then confirmed by carrying out qRT–PCR. Consistent with microarray data, increased expression of TGF-β1 and decreased expression of MUC4, and carbonic anhydrase 9 (CA-9) was found in MUC4-KD cells (Figure 5A). In a similar manner, up-regulation of CA-9, ITG-β6, ITG-β7 and TGF-β1 and down-regulation of ErbB2, and the calcium binding protein S100P was found in ErbB2-KD cells (Figure 5B).

## Discussion

The mucin MUC4 is thought to form a complex at the membrane with the oncogenic receptor ErbB2 based on biochemical studies with rat homologue of MUC4 [4], [13], [28]. Both MUC4 and ErbB2 are overexpressed in several epithelial cancers (pancreas, breast, lung, oesophagus, colon, ovary) and both have been shown to play roles in tumour development [12], [15], [28], [29], [30]. The potential of these two membrane proteins as therapeutic targets to slow down/stop tumour development is obvious and has drawn a lot of attention to develop tools targeting them. However, the direct interaction between human MUC4 and ErbB2 has not been proven at this time, moreover, studies on their role in regulating the biological properties of cancer cells have always been carried out separately in different cellular models [3], [15], [17]. Moreover, if they function as a complex, we must understand the molecular mechanisms that both proteins control in cancer cells in order to develop tools that will target the complex.

In this work, we have undertaken to study the biological consequences of both MUC4 and ErbB2 suppression in the same cellular model to determine whether they control the same signalling pathways to promote tumorigenesis and develop arguments in favour of an active MUC4-ErbB2 complex.

Our results clearly show that MUC4 and ErbB2 interact physically in CAPAN-2 cells and that they activate different signalling pathways, MUC4 activating the JNK pathway and ErbB2 activating the p42/44 MAPK pathway. We also show that they play roles in different properties of cancer cells with ErbB2 clearly affecting proliferation, cell cycle and tumour growth whereas MUC4 shows a wider spectrum of activities ranging from proliferation, cell cycle, tumour growth but also migration and invasion. This adds to the complexity of MUC4 biological activities in cancer since at least three major signalling pathways are under its control in pancreatic cancer cells. The fact that MUC4 affects a wider spectrum of biological activities may be explained by the fact that it not only forms a complex with ErbB2 and participates in downstream cell signalling [13], [28], [30] but also may have other partners at the membrane *via* its different modular domains [4], [29], [31]. Indeed, MUC4 may alter migration/invasion because of its huge glycosylated extracellular domain that has the capacity to interact with numerous kinds of lectins, selectins, and receptors. Alteration of mucin glycosylation is well-described in cancer [4], [29] and may modify MUC4 properties. Future investigations will be useful to better understand MUC4 biology related to its extracellular glycosylated domain as we found several glycosyl/fucosyl/sialyl-transferases with decreased (ST3GAL6, B3GNT5, B3GNT3, FUT8, FUT11) or increased (ST6GALNAC, FUT1) expression in our microarray data (table S3). Galectins, lectins that are known to interact with mucins and alter cancer cell properties [24], [32], [33], were found to be down-regulated in MUC4 deficient cells (galectin-1) (table S3). Recent work showed interaction between MUC4 and galectin-3 [34], thus interaction with the other galectins will have to be investigated in order to show whether these interactions are specific or more general. This illustrates the fact that the cellular context (environment) will have profound impacts on MUC4 biological role. This is especially true in pancreatic cancer where a huge stromal reaction surrounds epithelial counterparts of the tumour [35], [36], [37], [38]. Interestingly, we found downregulation of several components (integrins α1/α5/α6, epithelial-stromal interaction 1 (EPSTI1), fibronectin type III) that may, together with altered MUC4 expression in cancer cells, modify epithelial-stromal interactions. We also confirmed the strong link between MUC4, ErbB2 and ErbB3 [13] that excludes the two other members of the ErbB family (ErbB1 (EGF-R) and ErbB4), since ErbB2 silencing reduced ErbB3 and MUC4 suppression led to a reduction in expression of both ErbB2 and ErbB3.

**Figure 6:**
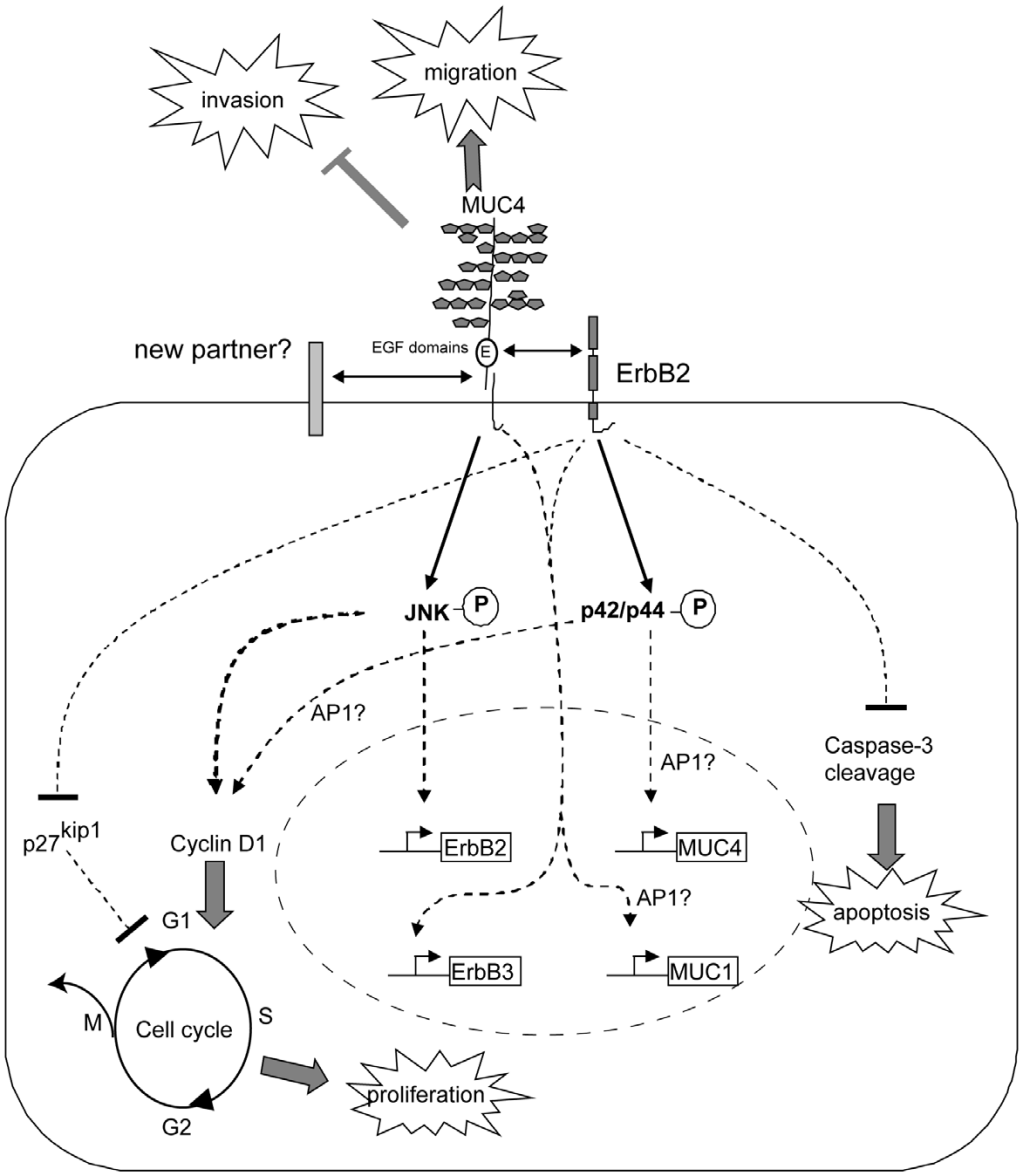
Schematic representation of ErbB2 and MUC4 distinct roles on biological properties of CAPAN-2 pancreatic cancer cells.

Decreased proliferation in cells lacking MUC4 is most likely due to the alteration of the JNK pathway since it is known that this pathway promotes proliferation and decreased apoptosis in a tumour context [39], two alterations that we found in MUC4-deficient cells. It is also clearly associated with cell cycle arrest, cyclin D1 repression as well as other members of cell cycle that we found decreased (>2 fold decrease) in microarray analyses (cyclin B1, cell division cycle 2 variant (CDC2), epithelial cell transforming sequence 2 oncogene (ECT2), a protein elevated during the G2-M phase of the cycle, cyclin G2) (table S3). Alteration of proliferation in cells lacking ErbB2 is clearly associated with alteration of the cell cycle and cyclin D1. The link between these pathways may be the activating protein-1 (AP-1), a transcription factor known to regulate MUC4 [40] and cyclin D1 [41] expression, the JNK pathway [42] and that is regulated by the p42/44 MAPK pathway [41]. We hypothesize that lack of ErbB2 may induce a reduction of p42/p44 activation and therefore a reduction of cyclin D1 and MUC4 expression *via* AP-1 (Figure 6). AP-1, *via* alteration of the JNK pathway, could also be responsible for the repression of the membrane-bound mucin MUC1 that was observed in MUC4-KD cells, since the *MUC1* promoter contains several binding sites for AP-1 (Figure 6). Concerted regulation of membrane-bound mucins has already been observed *in vivo* since downregulation of Muc1 mRNA was detected in the Muc16 homozygous knock-out mouse [43]. This could reflect a more general mechanism to counteract the absence of a membrane-bound mucin in certain situations and allow normal function of the cell [30], [44].

Another important pathway in pancreatic tumour progression is the TGF-β pathway that shows antagonistic effects on tumour cells as it represses proliferation in the early stages and promotes metastasis at later steps [45]. We previously showed that TGF-β1 is a key regulator of MUC4 expression in pancreatic cancer cells *via* the TGF-βRII/Smad3–4 pathway [20]. Accordingly, in MUC4 deficient cells we found, in microarray analyses, inhibition of Smad3 and TGF-βRII. We also found a strong induction of TGF-β1 mRNA expression in cells lacking ErbB2 or MUC4. From these results, we hypothesize that MUC4 inhibition induces expression of TGF-β_1_ at the mRNA level to create a loop of regulation (feedback) that would result in up-regulation of ErbB2 or MUC4 and promote tumour cell proliferation.

Despite the fact that it is thought that MUC4 may alter migration/invasion/adherence properties of cancer cells *via* its huge extracellular domain and modified steric hindrance [4], [29], adherence to two kinds of matrix was not modified in the absence of MUC4 in our model. On the other hand, the observed increased invasion in the absence of MUC4 was a bit surprising as we did not find any significant variation of metalloproteinase expression (MMP2, -7 or -9) (not shown) and decrease of collagen, type VIII alpha 1 (COL8A1) expression, a component of the stromal reaction in pancreatic cancer, was observed in microarray analyses. Previous data indicated that increased FAK phosphorylation and β-catenin levels in MUC4-transfected cells could influence cell migration without directly altering cell adhesion through its action on the ErbB2-ErbB3 complex [12], [15]. Our observation that MUC4 silencing leads to a decreased migration but no alteration of cell adhesion is in accordance with these data. The fact that FAK was not altered by MUC4 silencing in our model suggests that this property may be mediated by yet another pathway.

ErbB2 was not found to play major roles in cell migration/invasion as cells lacking ErbB2 did not show any significant alteration of migration/invasion despite the fact that we found a significant increase of integrin-β6 and -β7 expression in the transcriptome of ErbB2-KD cells. This may be explained by the fact that these two integrins are involved in migration/invasion in other cell types (keratynocytes) [46] or types of cancer (breast, colon, stomach and ovary) [47], [48], [49] and that in pancreatic cancer integrins β1/β4 seem more preponderant [50].

Finally, MUC4 activity on tumour cell properties is most likely cell-specific (state of differentiation, tumour site origin: primary or metastatic) as we did not find the same results with moderately differentiated CAPAN-2 cells, which derive from a primary tumour [51] when compared with previous studies conducted in the poorly differentiated pancreatic cancer cell model HPAF-CD18, which derives from peritoneal ascitic fluid [52].

In conclusion, we show that inhibition of ErbB2 and MUC4 expression did not impair the same signalling pathways (inhibition of MUC4 expression affects the JNK pathway whereas that of ErbB2 alters the MAPK pathway) and produced different effects on pancreatic cancer cell biological properties, suggesting independent activity of these two proteins (Figure 6). Our data also suggests a role in pancreatic tumour growth for ErbB2, while MUC4 is involved in both tumour growth and dissemination. These data bring new information regarding molecular mechanisms under the control of both MUC4 and ErbB2 that will have to be taken into account for developing efficient targeting of both proteins in order to slow down/stop pancreatic tumour development.

## Supporting Information

Figure S1 **Expression of MUC1 membrane-bound mucin and ErbB receptor family in the two representative clones of MUC4-KD and ErbB2-KD cells by Western blotting and their respective controls (Mock and NT).**
(TIF)

Figure S2 **Impact of ErbB2 and MUC4 on FAK and Akt signalling pathways.** Western blot were carried out for FAK, phospho-Akt and Akt in MUC4-KD, ErbB2-KD and their respective controls (Mock and NT). β-actin was used as the internal control.
(TIF)

Figure S3 **Venn diagram of regulated genes in MUC4-KD vs Mock cells compared with ErbB2-KD vs NT cells.**
(TIF)

Table S1 **Primer sequences used for qRT-PCR.**
(DOC)

Table S2 **List of gene differentially-regulated (fold change >2, p<0.05) in ErbB2-KD vs. NT control cells.**
(XLS)

Table S3 **List of gene differentially-regulated (fold change >2, p<0.05) in MUC4-KD vs. Mock control cells.**
(XLS)

Table S4 **Gene Ontology Analysis of ErbB2-KD vs. NT control cells.**
(XLS)

Table S5 **Gene Ontology Analysis of MUC4-KD vs. Mock control cells.**
(XLS)
