# Supplementary figures and images for: The Mucin MUC4 and Its Membrane Partner ErbB2 Regulate Biological Properties of Human CAPAN-2 Pancreatic Cancer Cells via Different Signalling Pathways
Source: PLoS One. 2012 Feb 29;7(2):e32232. doi: 10.1371/journal.pone.0032232 (PMC3290552; doi:10.1371/journal.pone.0032232)

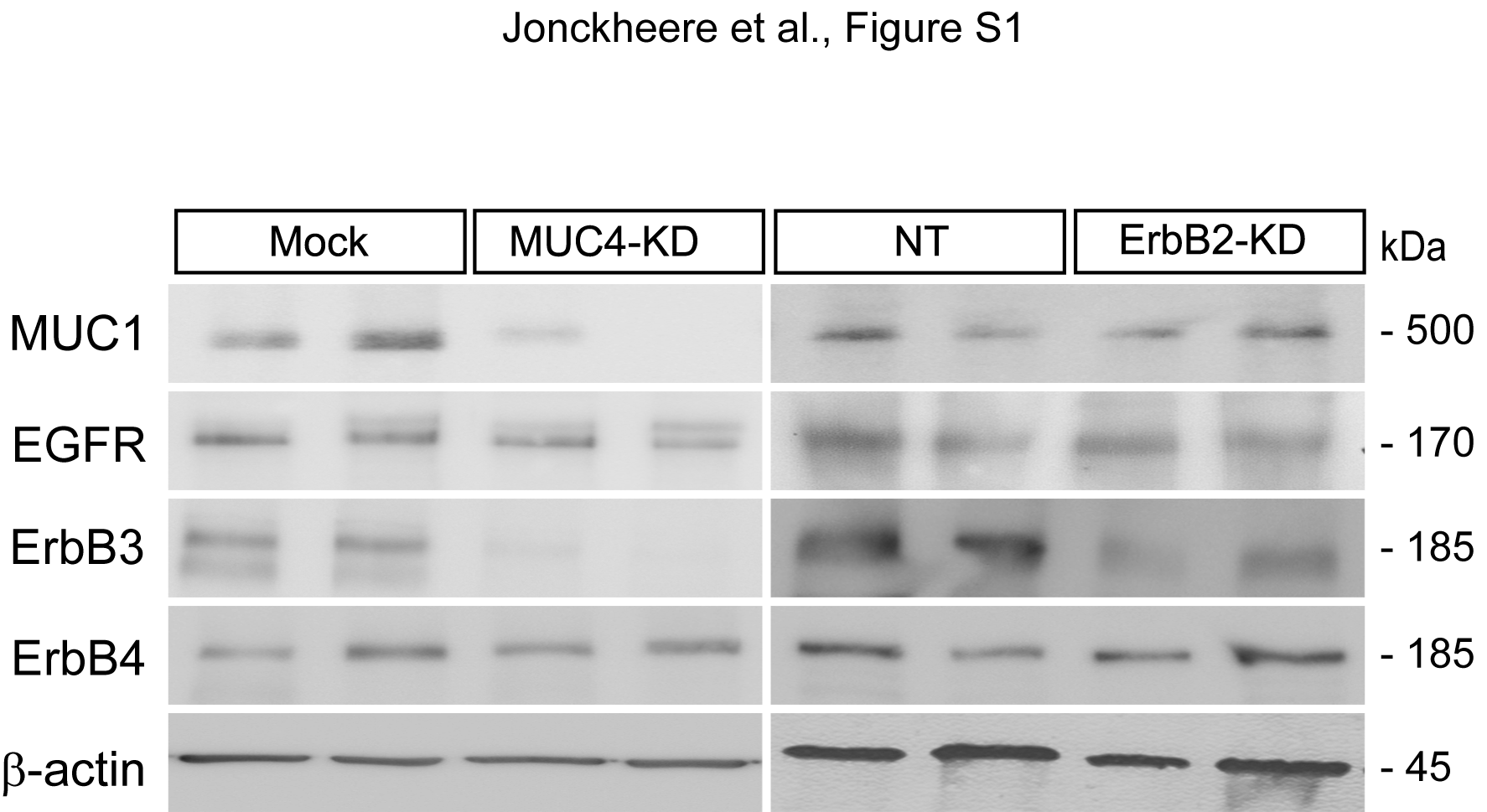

Supplement: Figure S1 — Expression of MUC1 membrane-bound mucin and ErbB receptor family in the two representative clones of MUC4-KD and ErbB2-KD cells by Western blotting and their respective controls (Mock and NT). (TIF) [file pone.0032232.s001.tif]

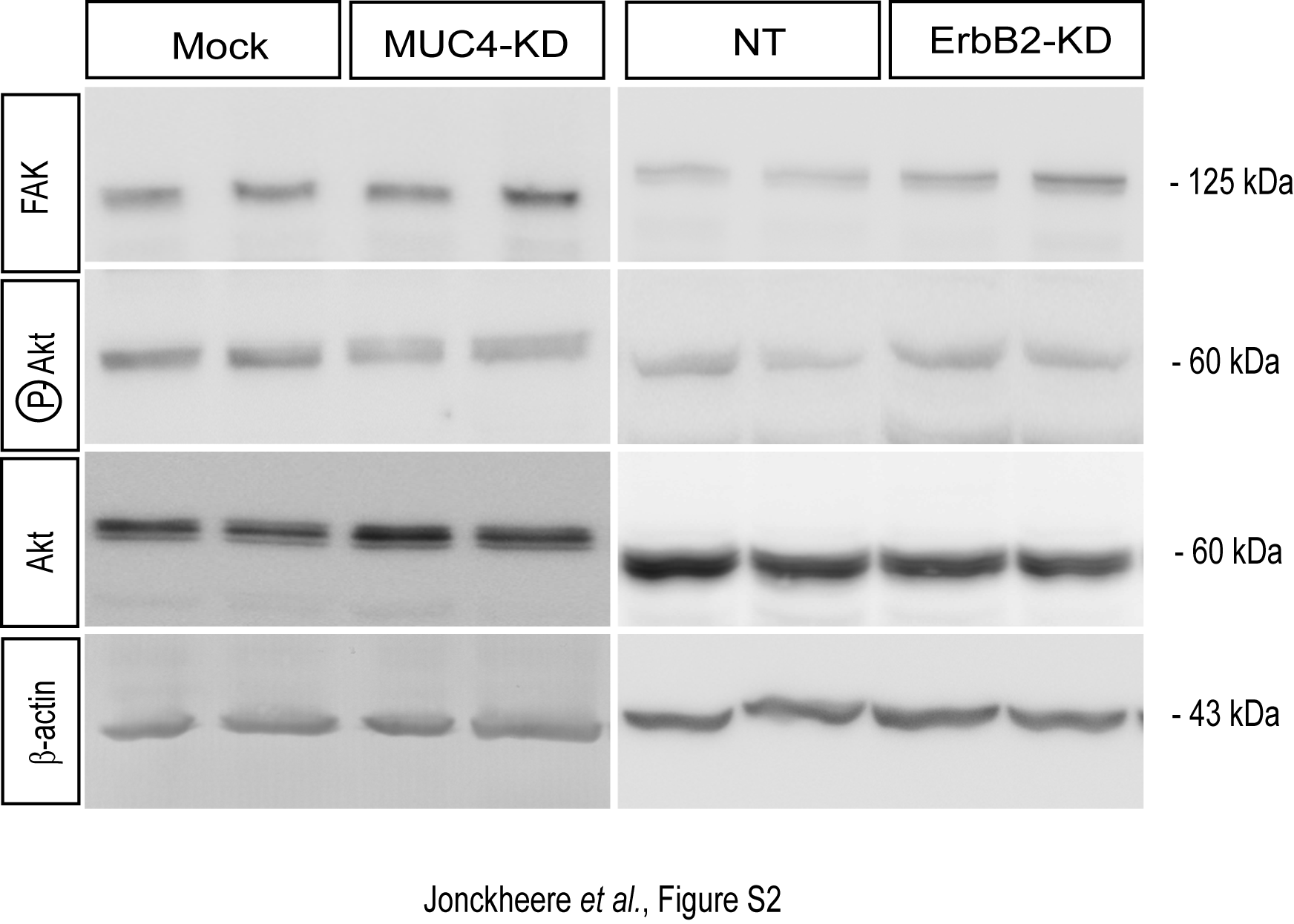

Supplement: Figure S2 — Impact of ErbB2 and MUC4 on FAK and Akt signalling pathways. Western blot were carried out for FAK, phospho-Akt and Akt in MUC4-KD, ErbB2-KD and their respective controls (Mock and NT). β-actin was used as the internal control. (TIF) [file pone.0032232.s002.tif]

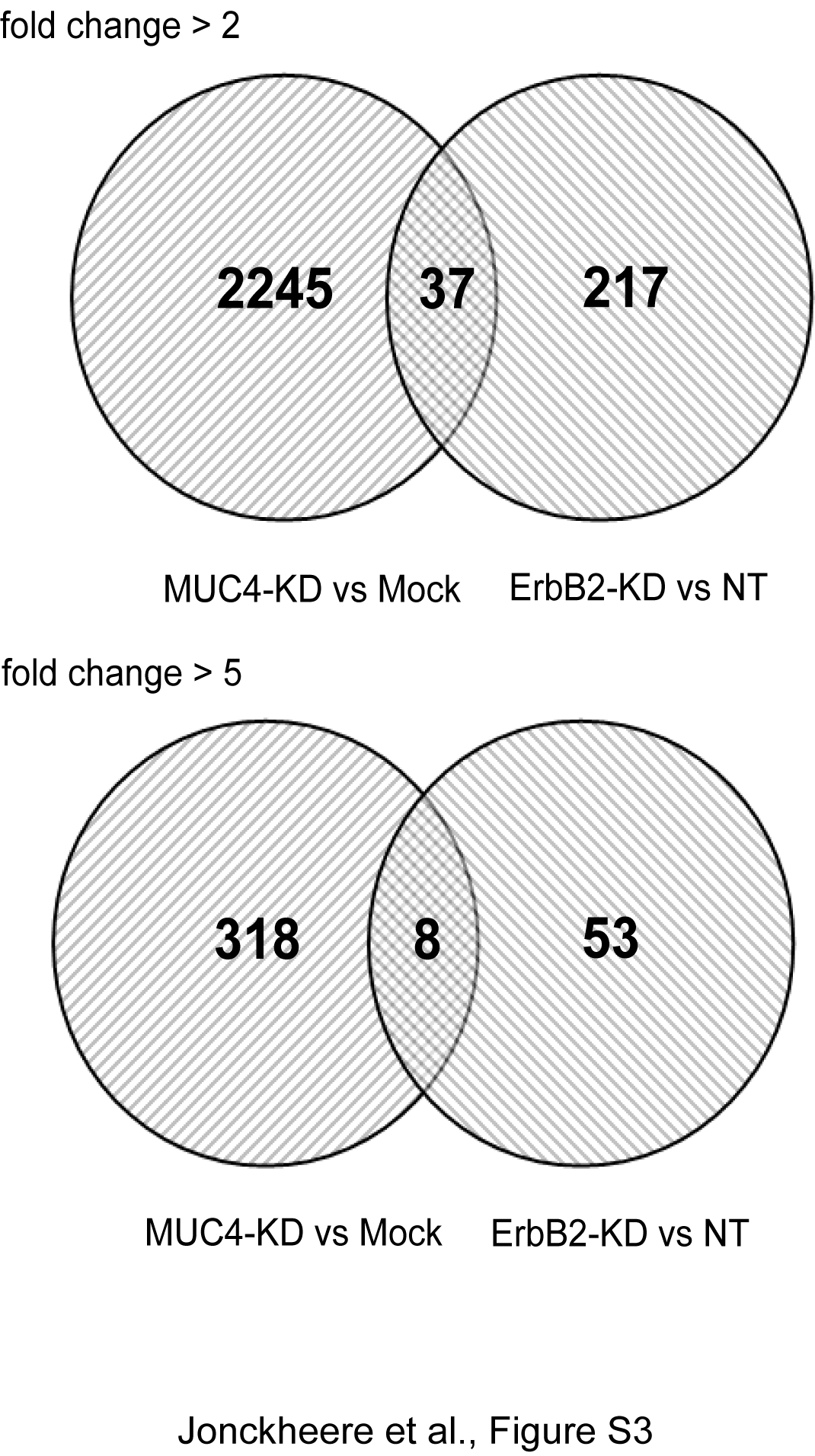

Supplement: Figure S3 — Venn diagram of regulated genes in MUC4-KD vs Mock cells compared with ErbB2-KD vs NT cells. (TIF) [file pone.0032232.s003.tif]
