# Supplementary material for: The Mucin MUC4 and Its Membrane Partner ErbB2 Regulate Biological Properties of Human CAPAN-2 Pancreatic Cancer Cells via Different Signalling Pathways
Source: PLoS One. 2012 Feb 29;7(2):e32232. doi: 10.1371/journal.pone.0032232 (PMC3290552; doi:10.1371/journal.pone.0032232)
Supplement: Table S1 — Primer sequences used for qRT-PCR. (DOC) [file pone.0032232.s004.doc]

| Gene | Primers (5’→3’) | Melting temperature(°C) |
| --- | --- | --- |
| MUC4 | F: GCC CAA GCT ACA GTG TGA CTC A  R: ATG GTG CCG TTG TAA TTT GTT GT | 50 |
| ErbB2 | R: CCA GCT GGC TCT CAC ACT G  F: AGC CCT TAC ACA TCG GAG AAC | 52 |
| S100P | F: AAA GAC AAG GAT GCC GTG GAT  R: AGG CAG ACG TGA TTG CAG C | 55 |
| ITGβ6 | F: CAT GTC CGC CAG ACT GAG G  R: GAG CCC AGC TCC TTT ATT GTG | 55 |
| ITGβ7 | F: ATG GTG GCT TTG CCA ATG GT  R : GGA CAG GTG AGG ATT CCG C | 55 |
| TGF-β1 | F: CAC TCT CAA ACC TTT ACG AGA CC  R: CGT TGC TAG GGG CGA AGA TG | 55 |
| CA9 | F: TTG CCA GAG TTG ACG AGG C  R: CGA TTT CTT CCA AGC GAG ACA G | 55 |
| GAPDH | F: CCA CAT CGC TCA GAC ACC AT  R: CCA GGC GCC CAA TAC G | 55 |

Table S1. : Primer sequences used for qRT-PCR.
